# *Sarcodon imbricatus* polysaccharides improve mouse hematopoietic function after cyclophosphamide-induced damage via G-CSF mediated JAK2/STAT3 pathway

**DOI:** 10.1038/s41419-018-0634-6

**Published:** 2018-05-21

**Authors:** Xue Wang, Qiubo Chu, Xue Jiang, Yue Yu, Libian Wang, Yaqi Cui, Jiahui Lu, Lirong Teng, Di Wang

**Affiliations:** 1 School of Life Sciences, Jilin University, Changchun, 130012 China; 2 Zhuhai College of Jilin University, Jilin University, Zhuhai, 519041 China

## Abstract

*Sarcodon imbricatus*, a rare medicinal and edible fungus, has various pharmacological bioactivities. We investigated the effects of *S. imbricatus* polysaccharides (SIPS) on hematopoietic function and identified the underlying mechanisms using in vitro experiments with CHRF, K562, and bone marrow mononuclear cells (BMMNCs) and in vivo experiments with a mouse model of cyclophosphamide-induced hematopoietic dysfunction. We found that SIPS induced proliferation and differentiation of CHRF and K562 cells and upregulated the expression of hematopoietic-related proteins, including p90 ribosomal S6 kinases (RSK1p90), c-Myc, and ETS transcription factor, in the two cell lines. After 28 days of treatment, SIPS enhanced the bodyweight and thymus indices of the mice, alleviated enlargement of the spleen and liver, and contributed to the recovery of peripheral blood to normal levels. More importantly, the percentages of B lymphocytes and hematopoietic stem cells or hematopoietic progenitor cells were significantly elevated in bone marrow. Based on an antibody chip analysis and enzyme-linked immunosorbent assay, SIPS were found to successfully regulate 12 cytokines to healthy levels in serum and spleen. The cytokines included the following: interleukins 1Ra, 2, 3, 4, 5, and 6, tumor necrosis factor α, interferon^−^γ, granulocyte colony-stimulating factor (G-CSF) and macrophage colony-stimulating factor (M-CSF), C-C motif chemokine1, and monocyte chemoattractant protein^−^1. Moreover, SIPS upregulated the phosphorylation levels of janus kinase 2 (JAK2) and the signal transducer and activator of transcription 3 (STAT3) in the spleen, and similar results were validated in CHRF cells, K562 cells, and BMMNCs. The data indicate that SIPS activated the JAK2/STAT3 pathway, possibly by interactions among multiple cytokines, particularly G-CSF. We found that SIPS was remarkably beneficial to the bone marrow hematopoietic system, and we anticipate that it could improve myelosuppression induced by long-term radiotherapy or chemotherapy.

## Introduction

Chemotherapy and radiotherapy are the main treatments for cancer, but they do not kill only cancer cells. They also destroy healthy cells^1^ or, worse, damage the hematopoietic system^2^. Radiation damage can trigger an oxidative stress imbalance^3^, endothelial cell aging^4^, aplastic anemia, or myelodysplastic syndrome^5^. Researchers have only partially explained the pathogenesis of bone marrow hematopoietic dysfunction caused by radiotherapy and/or chemotherapy, which can include (1) a lack of hematopoietic stem cells (HSCs) or an imbalance in the intrinsic cell cycle^6^; (2) bone marrow hematopoietic damage caused by a variety of hematopoietic cell growth factor secretion disorders^7^; or (3) cell or humoral immune system dysfunction^8^. Among these dysfunctions, a lack of HSCs or abnormalities in HSCs have been considered the main pathological mechanisms of hematopoietic dysfunction. Therefore, it is important to find a remedy that can effectively promote the recovery of hematopoietic function.

Granulocyte colony-stimulating factor (G-CSF), erythropoietin, or direct transfusion therapy are commonly used for hematopoietic dysfunction, but such treatments require frequent repetition^9,10^. Chemosynthetic myeloprotective agents, as an alternative treatment, are difficult to widely use clinically due to their inherent toxicity, which can damage bone marrow hematopoietic function and the bone marrow microenvironment over long-term use. Chemosynthetic myeloprotective agents also cause adverse reactions, such as peripheral leucopenia and myelosuppression^11^. Because of their pharmacologic properties and low level of adverse effects, effective active ingredients from herbs and/or fungi have recently been applied to promote recovery of hematopoietic function^12,13^. *Grifola frondosa* polysaccharides directly enhance the proliferation and differentiation of bone marrow cells into granulocytes-macrophages and protect the colony formation unit response of granulocytes-macrophages from doxorubicin-induced hematopoietic suppression^14^. *Angelica sinensis* polysaccharides ameliorate stress-induced premature senescence by protecting bone marrow stromal cells from chemotherapeutic injury, and further improve their hematopoietic function by mitigating oxidative damage to stromal cells^15^.

*Sarcodon imbricatus* (SI), is an edible and medicinal fungus that is widely distributed throughout Central Europe and North America^16^. Although SI has been anecdotally described as having various pharmacological effects, including anti-inflammation and anticancer activities, previous studies mainly focused on analysis of its chemical components and isolation of polysaccharides^16,17^. A water-soluble polysaccharide—a major component of SI—has been successfully isolated and its detailed structural features characterized^17^. Our group has studied the pharmacological activities of SI for years, and we discovered its improved immune function in cyclophosphamide (CTX)-induced immunosuppressive mice through an increase in interleukin (IL) 2 levels and regulation of oxidative stress^18^. However, the hematopoietic activities of SI polysaccharides and their underlying mechanisms have yet to be reported. In a hematopoietic microenvironment, a variety of cytokines form a highly complex and effective regulatory network to maintain the body’s normal hematopoietic function. IL-2 helps maintain erythropoiesis by modulating the activity of T cells (Treg) in the bone marrow^19^. In clinical conditions of bone marrow failure, IL-2 treatment might help restore hematopoiesis^20^. Because of IL-2’s important role in promoting bone marrow hematopoiesis and the link between immunity and hematopoiesis, we speculated that *S. imbricatus* polysaccharides (SIPS) could have positive effects on hematopoietic function.

We first investigated the effect of SIPS on the proliferation and differentiation activities of hematopoietic cells K562 and CHRF in vitro. We then used the hematopoietic dysfunction mouse model to explore the protective effect of SIPS on the hematopoietic system in vivo. To further reveal the role of SIPS in promoting hematopoietic recovery, we investigated the effects of the G-CSF-mediated janus kinase 2 (JAK2)/signal transducer and activator of transcription 3 (STAT3) signaling pathway.

## Results

### SIPS promoted proliferation and differentiation of hematopoietic cells

**Fig. 1:**
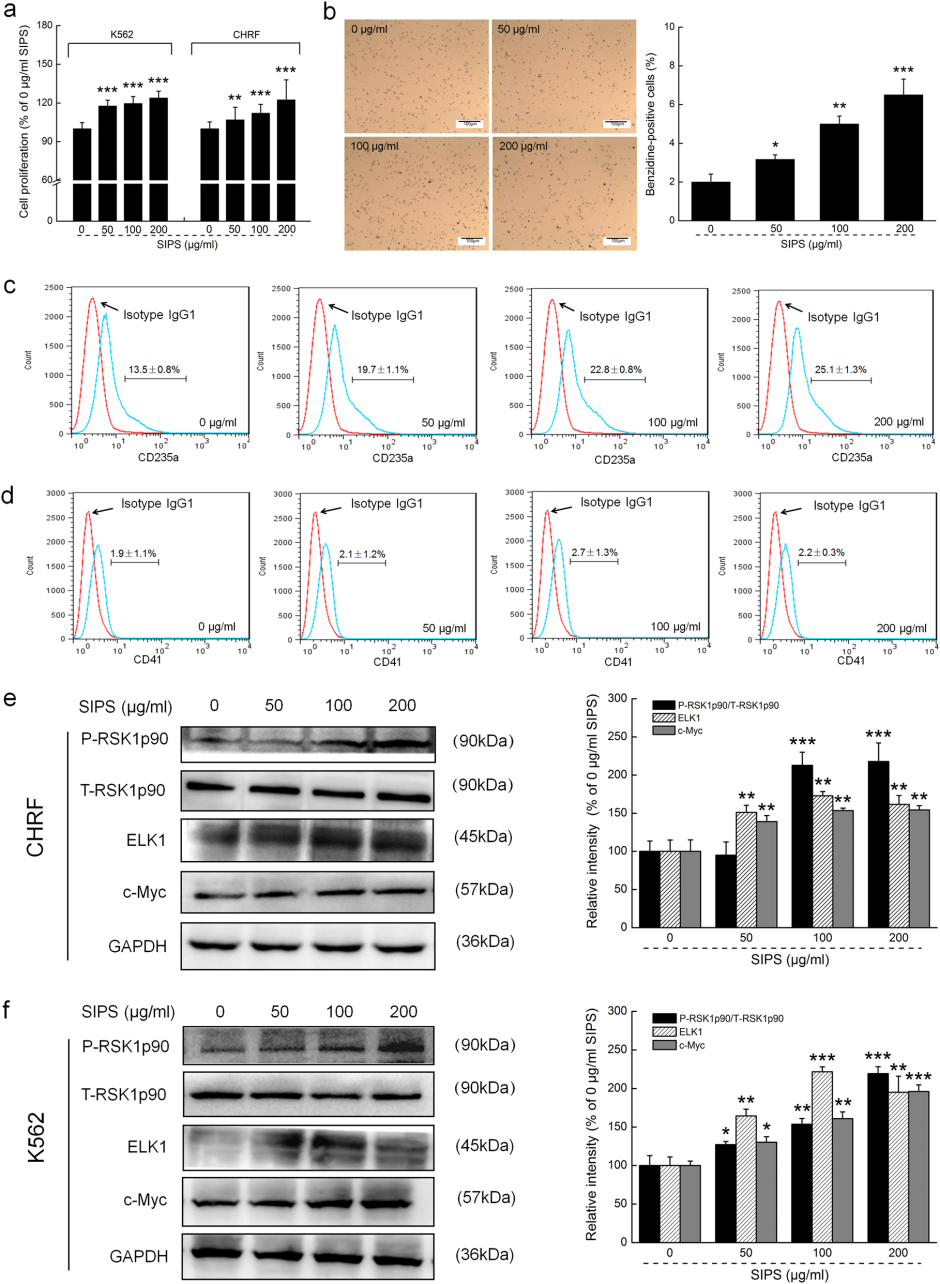
The potential properties of SIPS on the proliferation and differentiation of CHRF and/or K562 cells. Cells underwent a 24-h/48-h incubation with SIPS at doses of 0, 50, 100 and 200 μg/ml. **a** The cell proliferation of CHRF and K562 cells was analyzed using the XTT assay. **b** The erythroid differentiation of the K562 cells was analyzed by benzidine staining (10 × , scale bar: 100 μm). **c** The expression of glycophorin A (CD235a) and **d** integrin alpha 2b (CD41) in the K562 cell line was analyzed by flow cytometry, and the protein expression levels of P-RSK1p90, c-Myc and ELK1 in **e** the CHRF cells and **f** the K562 cells were detected by western blotting. The quantitative data of the protein expression levels were normalized by their GAPDH expressions and are shown as a percentage of the corresponding relative intensity of the control cells. Data are shown as the mean ± S.D. (*n* = 6). *P < 0.05, **P < 0.01 and ***P < 0.001 vs. 0 μg/ml SIPS-treated cells

Incubation with SIPS for 24 h strongly enhanced the proliferation of K562 and CHRF cells (*P* < 0.01; Fig. 1a). Benzidine staining combined with the expression of cell surface glycophorin A (CD235a) were applied, and we detected the erythroid differentiation of the K562 cells after SIPS exposure. In benzidine staining assays, the positive cell rate indirectly reflects the level of intracellular hemoglobin. Compared to nontreated cells, SIPS treatment resulted in *a* > 58% enhancement on cells positive for benzidine staining (*P* < 0.05; Fig. 1b). The expression level of CD235a reflects the progenitor’s differentiation ability into erythroid, and integrin alpha 2b (CD41) is the typical marker of megakaryocyte. Flow cytometry data showed that the expression levels of CD235a on the cell surface increased from 13.5% ± 0.8% to 25.1% ± 1.3% in the SIPS-treated K562 cells (*P* *<* 0.05; Fig. 1c). However, we found no significant changes in the CD41 levels in the SIPS-treated K562 cells (Fig. 1d). SIPS did not affect apoptosis in the K562 and CHRF cells (Fig. S2). The phosphorylation of RSK1p90 and the expression levels of c-Myc and ETS transcription factor in CHRF (*P* < 0.05; Fig. 1e) and K562 cells (*P* < 0.01; Fig. 1f) were strongly enhanced after incubation with SIPS for 24 h.

### Effects of SIPS on bodyweight and organ indices in mice with hematopoietic dysfunction

Compared to the control mice, the mice that received continual injections of CTX had a sharp decrease in bodyweight, splenomegaly, enlargement of the liver, and reduction of the thymus (*P* *<* 0.001; Table 1). The administration of SIPS and the injection of recombinant human granulocyte colony-stimulating factor (rhG-CSF) over 28 days increased the bodyweight and reversed the viscera lesions of mice with hematopoietic dysfunction (*P* *<* 0.05; Table 1). However, SIPS alone did not affect bodyweight or organ indices in healthy mice compared to control mice (*P* *>* 0.05; Table 1).

**Table.1:** The effects of SIPS and rhG-CSF on bodyweight and organ indexes

	Days	CTRL	CTX (100 mg/kg)					SIPS (50 mg/kg)
			--	rhG-CSF (22.5 μg/kg)	SIPS (25 mg/kg)	SIPS (50 mg/kg)	SIPS (100 mg/kg)	
Body weight (g)	1st day	24.9±2.1	24.1±1.2	24.1±1.6	25.2±1.7	24.5±0.8	24.8±1.9	25.2±1.8
	4th day	26.9±1.4	19.9±1.3^###^	19.5±1.5	20.8±1.9	19.9±1.7	20.2±1.9	26.3±2.4
	11th day	28.7±1.3	20.1±2.2^###^	22.8±1.6^*^	22.8±1.7^*^	22.1±1.3^*^	21.8±2.1^*^	27.3±2.5
	18th day	29.6±1.6	18.5±3.3^###^	22.6±1.4^**^	21.2±2.4^*^	21.8±2.1^*^	21.8±2.1^*^	28.6±1.2
	28th day	30.5±1.6	19.1±1.4^###^	23.8±1.1^**^	21.9±2.3^*^	23.5±2.5^**^	22.8±1.8^*^	29.6±2.4
Organ index (%)	Spleen index	4.1±0.7	12.3±3.8^###^	11.5±1.8	10.7±3.0	10.5±2.2^*^	9.9±2.5^*^	4.2±0.6
	Thymus index	1.9±0.3	1.2±0.4^###^	1.9±0.5^**^	1.8±0.4^*^	1.9±0.6^**^	1.7±0.3^*^	1.8±0.3
	Liver index	53.2±5.6	62.7±7.1^###^	59.4±3.8	60.4±4.4	58.9±3.2^*^	60.3±3.8	52.8±4.1
	Kidney index	15.6±1.2	16.1±1.0	16.7±0.9	16.2±1.1	16.5±1.2	16.2±1.1	16.1±1.0

Data are expressed as mean ± S.D. (*n* = 10/group)

*CTX* cyclophosphamide, *rhG-CSF* recombinant human granulocyte colony-stimulating factor, *SIPS* Sarcodon imbricatus polysaccharides

^###^*P* < 0.001 vs. control group, *P < 0.01 and **P < 0.01 vs. model group

### SIPS increased the quantity of peripheral blood cells and the production of murine bone marrow cells

The number of blood cells in the peripheral blood can indirectly indicate the hematopoietic function of bone marrow. We found that the levels of neutrophils, lymphocytes, hemoglobin, average erythrocyte hemoglobin content, and mean erythrocyte hemoglobin concentration in CTX-treated group were significantly lower than those of the control group (*P* *<* 0.05; Table 2). However, administration of 25, 50, and 100 mg/kg doses of SIPS over 28 days reversed this decrease, indicating that SIPS co-administered with CTX could increase leukocyte levels more than CTX alone.

**Table.2:** The effects of SIPS on peripheral blood cells of CTX-injected mice with hematopoietic injury

	CTRL	CTX (100 mg/kg)					SIPS (50 mg/kg)
		--	rhG-CSF (22.5 μg/kg)	SIPS (25 mg/kg)	SIPS (50 mg/kg)	SIPS (100 mg/kg)	
NE (%)	19.0±3.1	14.1±2.2^#^	31.1±3.9^**^	22.2±1.5	24.5±2.8^*^	28.4±3.3^**^	20.9±1.9
LY (%)	49.9±2.9	22.1±2.1^##^	31.3±1.1^**^	39.6±2.0^**^	26.8±3.8^*^	29.6±5.3^*^	50.8±8.6
MO (%)	28.7±1.6	66.7±5.2^###^	34.4±2.3^*^	39.1±2.7^**^	36.7±7.5^*^	33.1±6.3^*^	27.4±5.8
HGB (g/L)	146.0±8.2	128.5±2.5^##^	130.5±5.5	136.7±13.8^*^	134.0±3.0^*^	134.5±3.5^*^	138.0±10.6
MCV (fL)	43.5±1.8	41.8±0.8	40.8±1.6	44.0±1.8	42.8±0.5	43.3±2.1	42.9±1.2
MCH (pg)	14.8±0.6	13.6±0.2^#^	15.9±1.5^*^	14.5±1.7	14.8±1.2^*^	14.6±0.9	14.1±0.2
MCHC (g/L)	340.3±3.3	302.5±6.5^##^	330.3±4.5	330.7±3.7	333.7±6.5	336.7±7.1^*^	329.3±6.6
PLT ( × 10^9^/L)	710.7±105.4	1233.0±120.3^##^	1037.0±36.3^*^	1104.3±107.4	898.0±175.4^**^	1063.0±95.2	736.0±94.0

Data are showed as the means ± S.D. (*n* = 10/group) and analyzed using a one-way analysis of variance followed by Dunn’s test

*CTX* cyclophosphamide, *rhG-CSF* recombinant human granulocyte colony-stimulating factor, *SIPS* *Sarcodon imbricatus* polysaccharides

^#^*P* < 0.05, ^##^*P* < 0.01 and ^###^ *P* < 0.001 vs. control group, *P < 0.05 and **P < 0.01 vs. model group

**Fig. 2:**
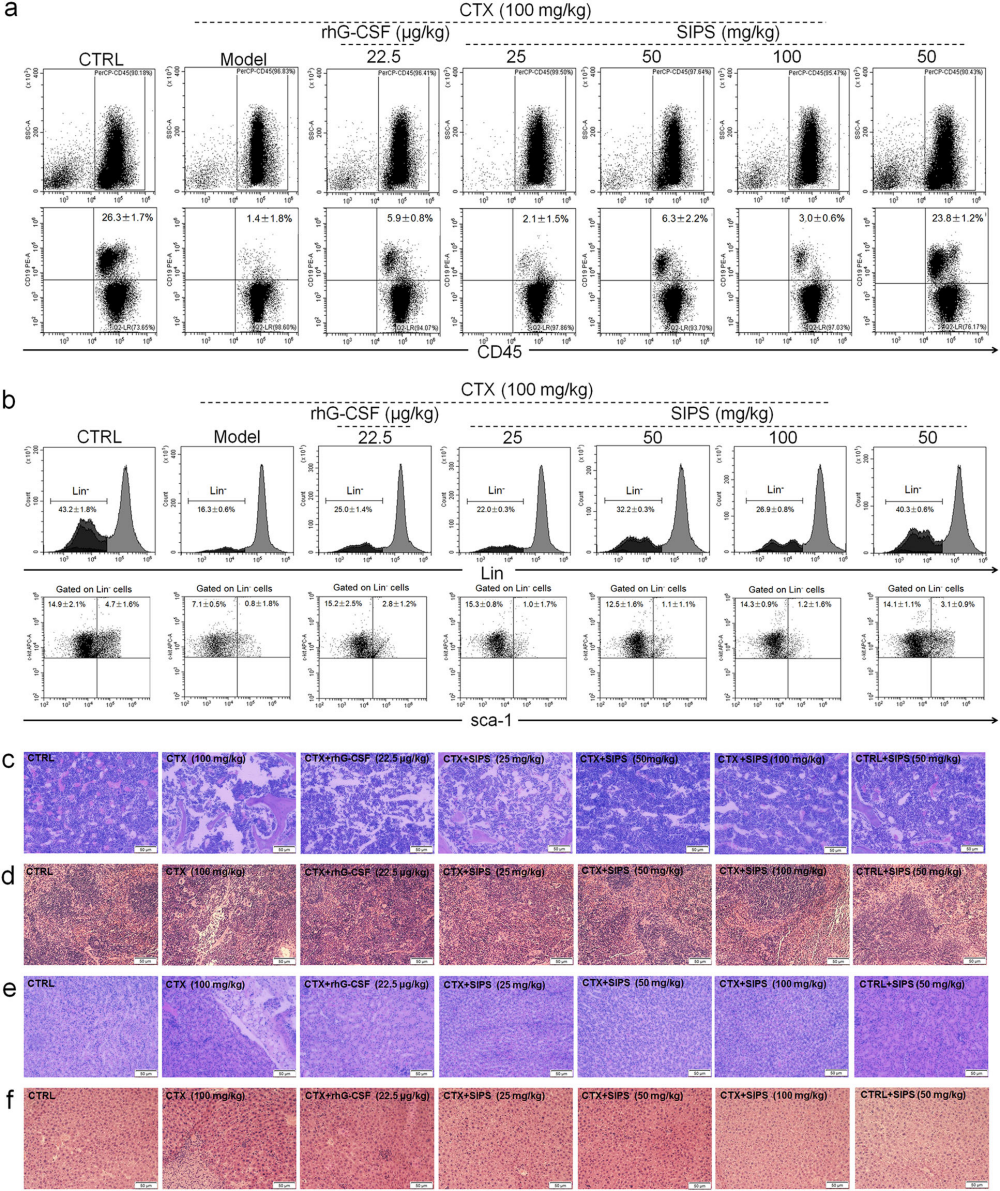
SIPS (25, 50, and 100 mg/kg) and rhG-CSF (22.5 μg/kg) enhanced the CTX (100 mg/kg)-induced decrement of leukocytes, HSCs/HPCs, and total bone marrow cellularity production in the murine bone marrow of mice after administration for 28 days. **a** Flow cytometry was used to analyze the proportion of leukocytes in murine bone marrow. CD45 was used for the sorting of leukocytes. CD45^+^ CD19^+^ represents the B lymphocytes. **b** The percentage of HSCs (Lin^-^c-kit^+^sca-1^+^) and HPCs (Lin^-^c-kit^+^sca-1^-^) in murine bone marrow of the CTX-injected mice were analyzed using a flow cytometry assay. The H&E staining procedure was used to evaluate the pathological alterations. **c** The cellularity of bone marrow; **d** spleen; **e** kidney; and **f** liver under a light-microscope digital camera (20 × , scale bar: 50 μm)

CD45 is a common leukocyte antigen expressed on all leukocytes. We used CD45^+^CD19^+^ as a surface marker to characterize the B lymphocytes phenotype of bone marrow mononuclear cells (BMMNCs). In the control group, 26.3% of CD45^+^CD19^+^ positive cells were noted in the BMMNCs, which decreased to 1.4% in BMMNCs isolated from mice with hematopoietic dysfunction (*P* *<* 0.001; Fig. 2a and Fig. S3a). The administration of both SIPS and rhG-CSF over 28 days caused enhancement of CD45^+^CD19^+^-positive cells in BMMNCs (*P* *<* 0.05; Fig. 2a and Fig. S3a).

We measured the specific expression of sca-1 and c-kit, which are screening markers for HSCs and hematopoietic progenitor cells (HPCs), to characterize the differentiation potential of SIPS on bone marrow cells. We quantified Lin^-^c-kit^+^sca-1^+^ and Lin^-^c-kit^+^sca-1^−^ cells to assess the percentages of HSCs and HPCs, respectively, in the total BMMNCs. The numbers of HSCs and HPCs in the hematopoietic dysfunction model group were 0.8 and 7.1%, respectively. Both numbers were significantly less than those for the control group (HSCs median, 4.7%, HPCs median, 14.9%; *P* *<* 0.01; Fig. 2b, S3b, and S3c). After administration of SIPS and rhG-CSF over 28 days, the counts of Lin^−^c-kit^+^sca-1^+^ and Lin^−^c-kit^+^sca-1^−^ cells were 1.2 and 15.3%, respectively (*P* *<* 0.05, Fig. 2b, S3b, and S3c).

A pathological examination was performed to explore the effect of SIPS on the hematopoietic recovery function of bone marrow cellularity and organs. Hematoxylin and eosin (H&E) staining showed that the density of the cells in the bone marrow cavity of the control mice was evenly distributed and neatly arranged. The number of cells in the bone marrow cavity of mice with hematopoietic dysfunction decreased, this was accompanied by the appearance of vacuoles (Fig. 2c). Multinucleated giant cells and slight extramedullary hematopoiesis appeared in the spleens of CTX-treated mice (Fig. 2d). Liver damage in mice with hematopoietic dysfunction was suggested by the inflammatory infiltration phenomenon and slight extramedullary hematopoiesis (Fig. 2e). SIPS reversed the CTX-induced hematopoietic dysfunction, as shown by the recovery of cell numbers in the marrow cavity, the reduction of the proportion of vacuoles (Fig. 2c), the simultaneous digestion of excessive multinucleated giant cells in the spleen (Fig. 2d), and the reduction of inflammatory infiltration in the liver (Fig. 2f). We found no significant difference in the structure of the kidneys in any experimental mice (Fig. 2e). Compared to the healthy control mice, the experimental mice treated with SIPS alone showed no differences in the number of peripheral blood cells (*P* *>* 0.05; Table 2) or the production of BMMNCs (Fig. 2a,b). The pathological features of the marrow cavity (Fig. 2c), spleen (Fig. 2d), kidney (Fig. 2e), and liver (Fig. 2f) also did not differ from those of the healthy control mice.

### SIPS regulated hematopoietic cytokine levels in mice serum and spleen

**Fig. 3:**
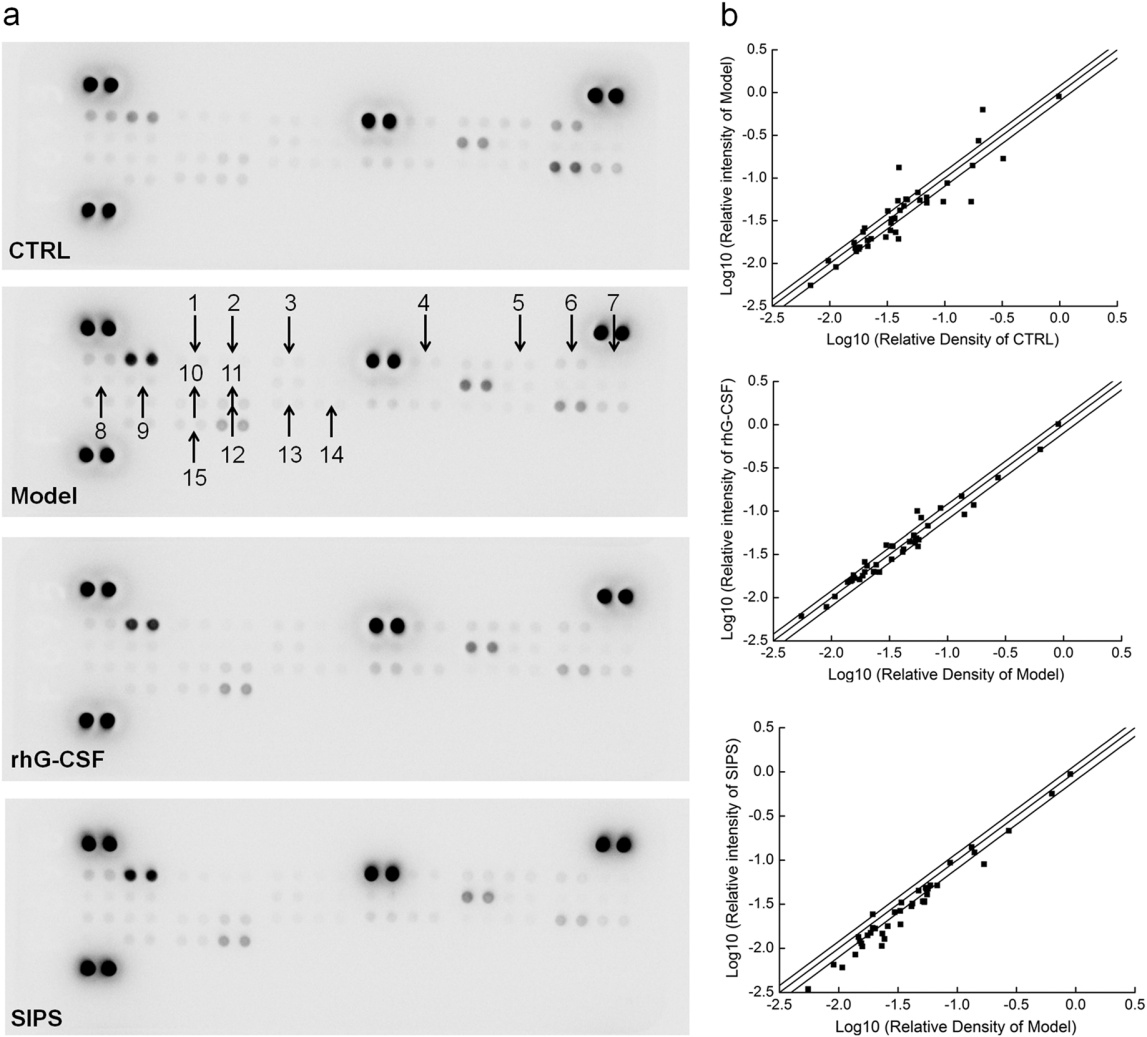
The effects of SIPS (50 mg/kg) and rhG-CSF (22.5 μg/kg) on the 40 cytokines in mice spleens were detected by a Mouse Cytokine Array Panel A Kit. **a** Graphical representation of cytokine expressions. The arrows indicate the factors with a change of >20% (SIPS group vs. model group). 1. G-CSF; 2. GM-CSF; 3. CCL1; 4. IFN- γ; 5. IL-1β; 6. IL-1Ra; 7. IL-2; 8. IL-3; 9. IL-4; 10. IL-5; 11. IL-6; 12. M-CSF; 13. MCP-1; 14. MCP-5; 15. TNF-α; **b** Scatter diagram of the 40 cytokines. The relative density is the ratio of the absolute value and the reference spot value

A Mouse Cytokine Array Panel A Kit was used to detected 40 cytokines related to the immune system in the spleens of mice with hematopoietic dysfunction that were administered SIPS for 28 days at a dose of 50 mg/kg. We found that the SIPS treatment strongly regulated the levels of 15 cytokines in the spleen (Fig.3). IL-2, IL-3, and IL-5 played important roles in the survival, proliferation, and differentiation of the pluripotent HSCs. Other cytokines, such as IL-1Ra, IL-4, and IL-6, acted as cofactors in the process. Based on a high-throughput splenic antibody chip analysis, we used an enzyme-linked immunosorbent assay kit to detect the contents of ILs, including IL-1Ra, IL-2, IL-3, IL-4, IL-5, and IL-6 in serum and in the spleen. CTX reduced the levels of all chosen interleukins except IL-1Ra in serum and in the spleen (*P* *<* 0.05; Table 3). In contrast, SIPS elevated the levels of IL-2, IL-3, IL-4, IL-5, and IL-6, and decreased the levels of IL-1Ra to the normal horizon in serum and in the spleen, compared to CTX-treated mice with hematopoietic dysfunction (*P* *<* 0.05; Table 3).

**Table.3:** The effects of SIPS on interleukins of CTX-injected mice with hematopoietic dysfunction

		CTRL	CTX (100 mg/kg)					SIPS (50 mg/kg)
			--	rhG-CSF (22.5 μg/kg)	SIPS (25 mg/kg)	SIPS (50 mg/kg)	SIPS (100 mg/kg)	
Serum	IL-1Ra	57.1±4.9	62.9±6.6^#^	49.1±4.5^*^	49.6±6.5^*^	55.6±4.2	42.3±4.1^**^	48.4±6.1^#^
	IL-2	2515.4±283.0	1964.5±174.1^#^	1708.9±122.4	2170.9±193.1	2309.3±103.8^*^	2320.9±304.6^*^	2166.5±249.8
	IL-3	45.0±3.7	38.8±2.1^##^	61.2±6.6^***^	45.4±4.3^**^	54.0±7.4^***^	59.0±7.1^***^	43.0±6.3
	IL-4	75.8±8.3	66.8±7.5^#^	93.2±11.4^***^	77.2±8.0^*^	85.3±6.8^**^	83.9±13.0^**^	73.8±10.2
	IL-5	21.2±2.2	12.1±2.4^###^	19.4±2.9^**^	17.7±1.4^*^	19.7±1.8^**^	15.1±2.7	22.8±3.3
	IL-6	74.7±9.6	59.3±5.6^#^	48.2±5.5^*^	67.4±2.2^*^	70.9±9.0^*^	68.1±4.8^*^	67.5±4.6
Spleen	IL-1Ra	27.7±4.1	36.1±4.6^#^	32.6±4.8	33.8±5.1	32.6±3.9	27.9±4.4^*^	29.9±5.4
	IL-2	923.9±44.4	855.1±63.3^#^	1054.9±128.4^**^	1023.2±60.1^*^	1055.6±106.4^**^	1021.6±76.3^*^	877.9±52.2
	IL-3	21.3±2.1	17.9±0.5^#^	16.2±1.8	17.4±2.3	18.5±1.6	18.4±2.0	22.1±2.3
	IL-4	37.4±4.1	30.2±5.3^#^	38.6±4.4	38.2±3.1	36.6±2.7	40.9±3.1^*^	38.5±6.1
	IL-5	6.0±1.0	3.4±1.2^##^	6.0±0.8^**^	5.6±0.6^**^	5.7±1.2^*^	4.9±0.5^*^	5.2±0.4
	IL-6	62.7±4.6	52.4±6.6^#^	60.6±3.1	69.9±7.6^*^	58.5±1.4	61.1±4.5	63.8±4.4

Data are expressed as mean ± S.D. (*n* = 10/group) and analyzed using a one-way analysis of variance followed by Dunn’s test

*CTX* cyclophosphamide, *rhG-CSF* recombinant human granulocyte colony-stimulating factor, *SIPS* Sarcodon imbricatus polysaccharides

^#^*P* < 0.05, ^##^*P* < 0.01 and ^###^*P* < 0.001 vs. control group, *P < 0.05, **P < 0.01 and ***P < 0.001 vs. model group

**Fig. 4:**
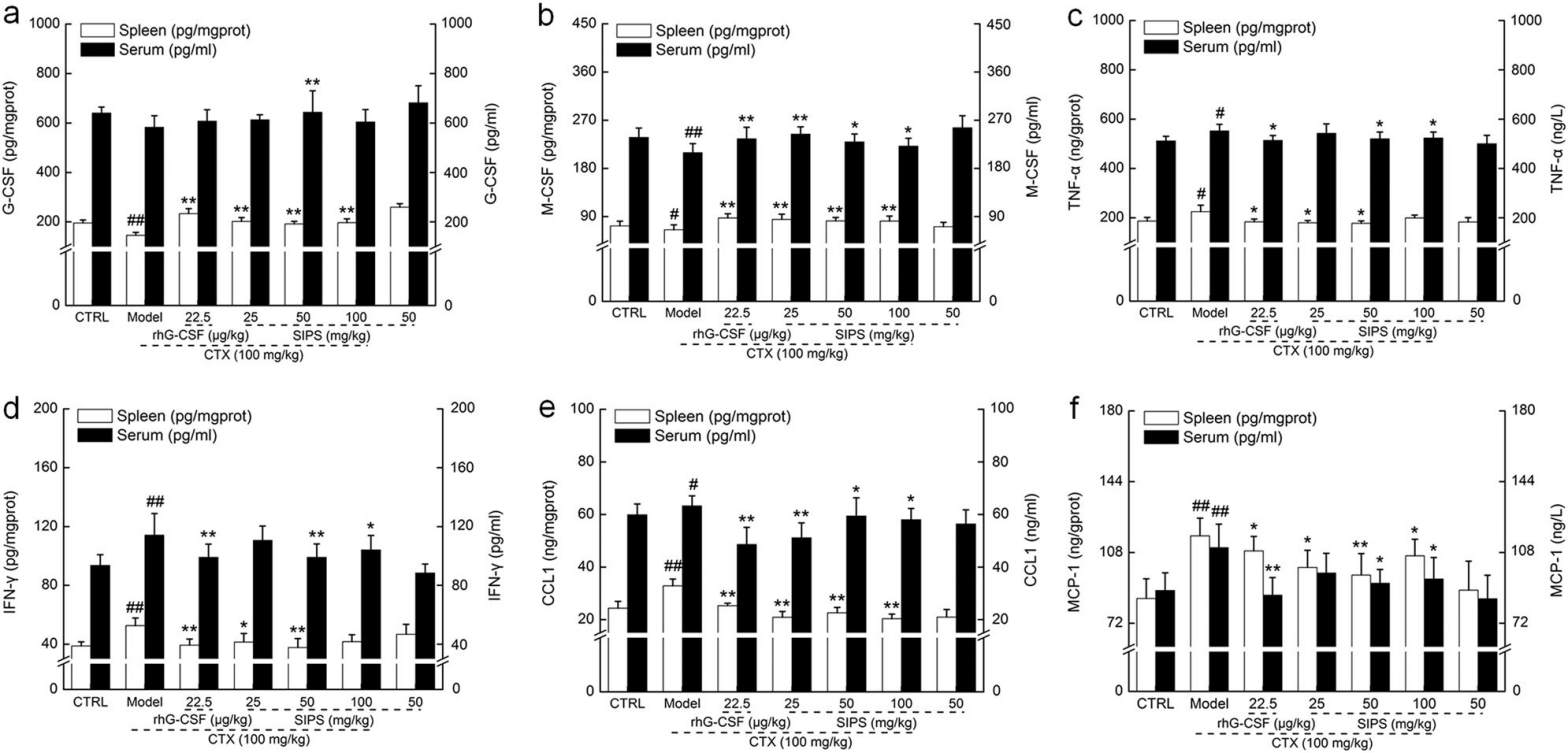
After a 3-day injection with CTX (100 mg/kg), a 28-day treatment with SIPS (25, 50, and 100 mg/kg) and a rhG-CSF treatment (22.5 μg/kg), the levels of six factors related to hematopoietic function in the serum and spleen of mice were detected using the ELISA method: **a** G-CSF, **b** M-CSF, **c** TNF-α, **d** IFN-γ, **e** CCL1, and **f** MCP-1. The data are expressed as the mean ± S.D. (*n* = 10). ^#^*P* < 0.05 and ^##^*P* < 0.01 vs. control group, *P < 0.05 and **P < 0.01 vs. model group

G-CSF and macrophage colony-stimulating factor (M-CSF), which are well known hematopoietic growth factors, can stimulate bone marrow to produce granulocytes and promote the proliferation and maturation of HSCs. Negative regulators secreted by T cells, tumor necrosis factor-α (TNF-α), and interferon gamma (IFN-γ) can act directly on HPCs to inhibit erythrocyte hematopoiesis or act indirectly by inducing the secretion of other cytokines. C–C motif chemokine (CCL1) and monocyte chemoattractant protein-1 (MCP-1) usually inhibit the proliferation of myeloid progenitor cells under the combined effects of various growth factors^21^. Studies have found that when synergized with transforming growth factor-β, MCP-1 could inhibit the growth of naive normal progenitor cells in a long-term culture^22^. Therefore, we analyzed the ongoing levels of G-CSF, M-CSF, TNF-α, IFN-γ, CCL1, and MCP-1 in serum and in the spleen. In CTX-treated mice, we found reduced levels of G-CSF and M-CSF and increased levels of TNF-α, IFN-γ, CCL1, and MCP-1 in serum and in the spleen (*P* *<* 0.05; Fig. 4). Treatment with SIPS over 28 days enhanced the levels of G-CSF and M-CSF in the serum and the spleens of mice with hematopoietic dysfunction (*P* *<* 0.05; Fig. 4a, b). However, the rhG-CSF treatment showed no significant effects on the levels of G-CSF in the spleen (Fig. 4a). Both SIPS and rhG-CSF significantly suppressed the hyper-levels of TNF-α, IFN-γ, CCL1, and MCP-1 in the serum and spleen of CTX-injected mice (*P* *<* 0.05; Fig. 4c–f). Compared to the control mice, SIPS alone showed no significant effects on the levels of all detected cytokines in the serum and spleen of healthy mice (Fig. 4a-f). SIPS had no significant effect on the other three cytokines (*P* *>* 0.05; Table S2).

### SIPS regulated the expression of the G-CSF-mediated STAT3 pathway and related proteins

**Fig. 5:**
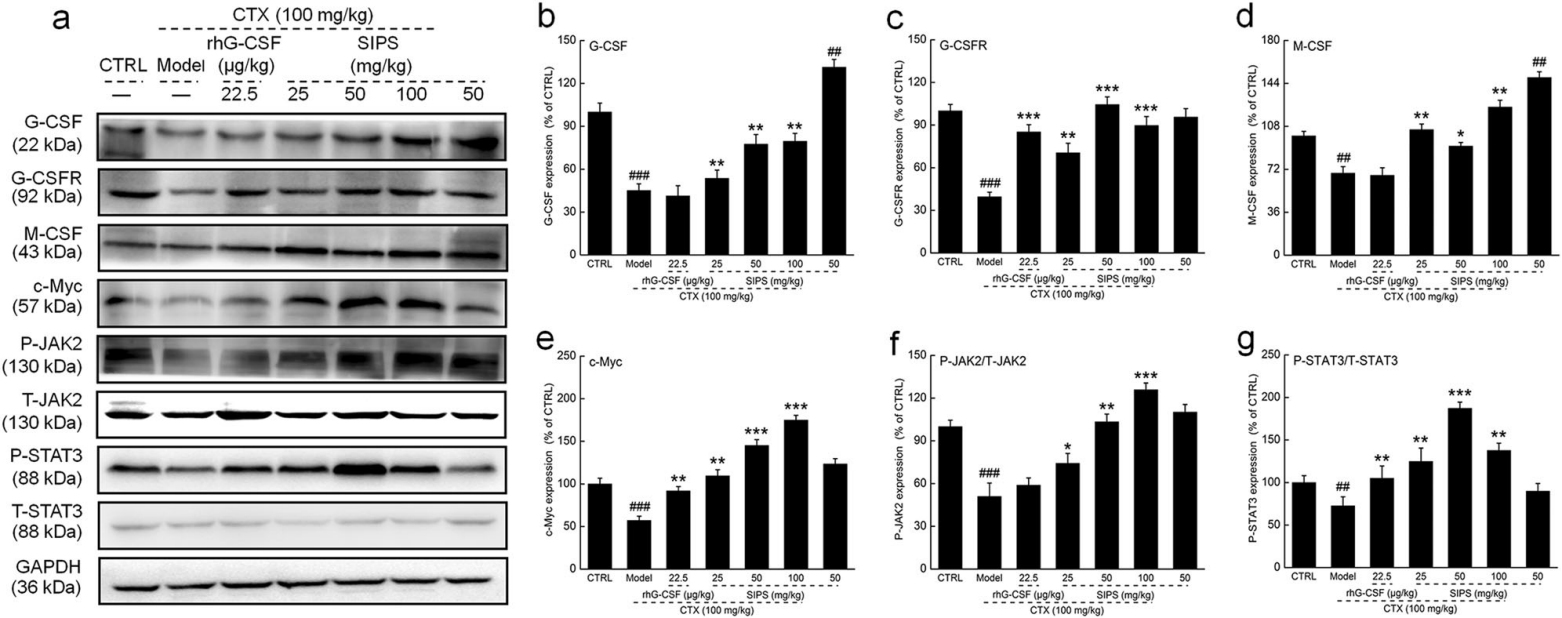
The G-CSF-mediated JAK2/STAT3 signaling pathway is, at least, partially involved in the protective effects of SIPS on mice with CTX-induced hematopoietic dysfunction. (**a**) SIPS (25, 50, and 100 mg/kg) enhanced the levels of G-CSF, G-CSFR, M-CSF, P-JAK2, P-STAT3, and c-Myc in the spleen of CTX-injected mice. The quantitative data of the **b** G-CSF, **c** G-CSFR, **d** M-CSF, **e** c-Myc, **f** P-JAK2, and **g** P-STAT3 levels were normalized by the corresponding GAPDH and related total protein expressions. Values are the percentage of the corresponding relative intensity of the control. The data are shown as the mean ± S.D. (*n* = 10). ^##^*P* < 0.01 and ^###^*P* < 0.001 vs. control group, *P < 0.05, ** *P* *<* 0.01 and *** *P* *<* 0.001 vs. model group

STAT3 signaling not only plays a key role in the growth of HSCs, but also affects their differentiation^23,24^. We analyzed the expression levels of proteins within the G-CSF–mediated STAT3 signaling pathway. We found extremely low expression levels of G-CSF, G-CSFR receptor, M-CSF, and c-Myc in the spleen tissues of CTX-treated mice (*P* *<* 0.001; Fig. 5). Comparatively, SIPS upregulated the spleen expression levels of G-CSF by more than 18.8%, G-CSF receptor by more than 78.3%, M-CSF by more than 32.9%, and c-Myc by more than 91.6% (*P* < 0.05, Fig. 5b–e). Moreover, compared to CTX-treated mice, SIPS enhanced the phosphorylation levels of JAK2 by more than 45.4% and STAT3 by more than 65.0% (*P* < 0.05, Fig. 5f, g).

**Fig. 6:**
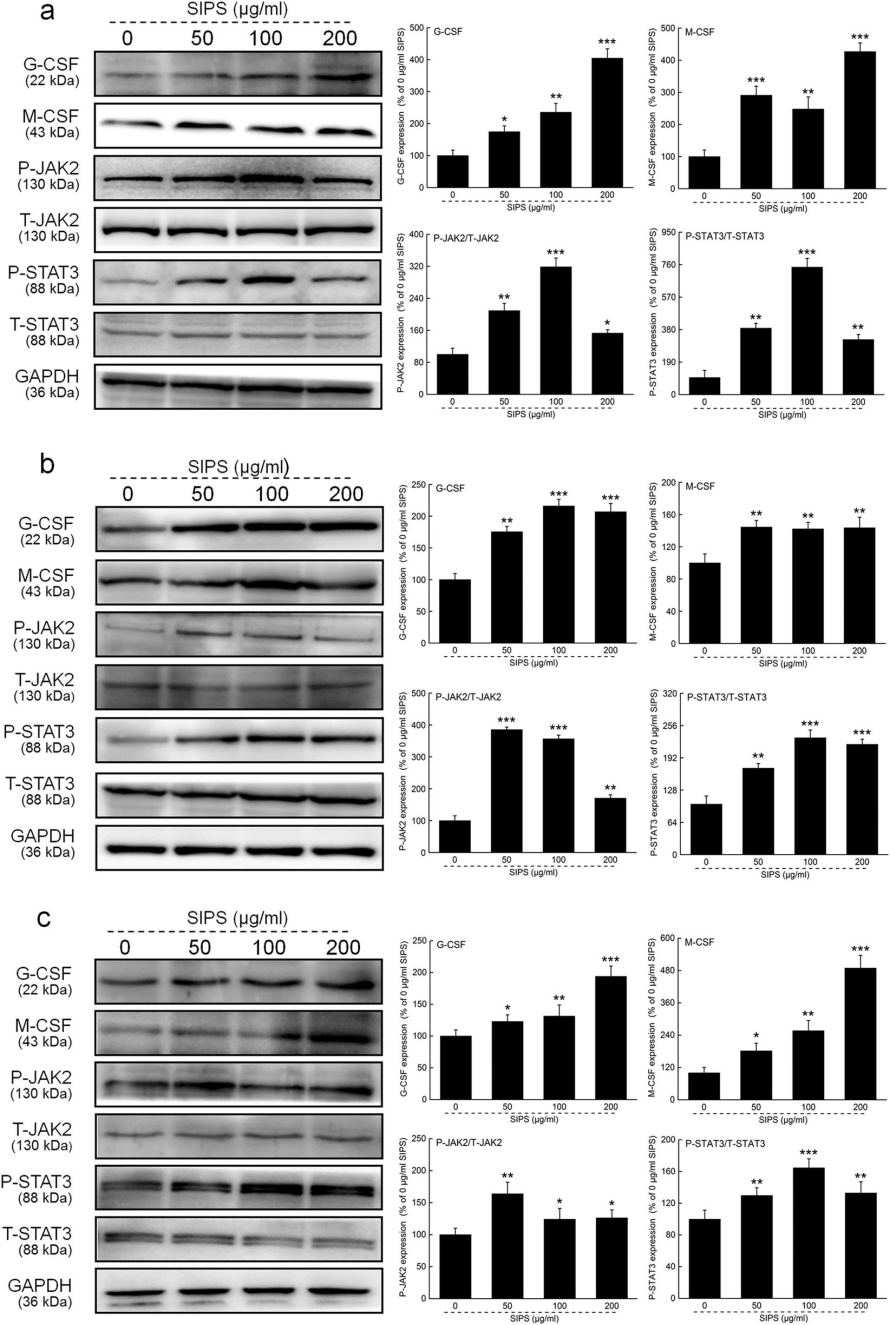
The G-CSF-mediated JAK2/STAT3 signaling was promoted by SIPS in CHRF, K562 cell and primary cultured BMMNCs. After 24-h incubation with SIPS, the protein expression levels of G-CSF, M-CSF, P-JAK2, P-STAT3 in CHRF cells (**a**), K562 cells (**b**) and BMMNCs (**c**) were analyzed by western blotting. The protein levels were normalized by the corresponding GAPDH and related total protein expressions. The values are the percentage of the corresponding relative intensity of the control. Data are shown as the mean ± S.D. (*n* = 10). * *P* < 0.05, ** *P* < 0.01 and *** *P* < 0.001 vs. 0 μg/ml SIPS-treated cells

We used a similar procedure in CHRF, K562 cell line, and primary cultured BMMNCs, and the expression levels of G-CSF, M-CSF, P-JAK2, and P-STAT3 were detected after 24-h incubation with SIPS. As expected, SIPS significantly upregulated the expression levels of the four proteins compared to the non–SIPS-treated CHRF cell line (*P* < 0.05, Fig. 6a), K562 (*P* < 0.01, Fig. 6b), and the BMMNCs (*P* < 0.05, Fig. 6c).

## Discussion

Acute myelosuppression results from the apoptosis of rapidly proliferating HPCs. Long-term bone marrow damage is mainly due to loss of the self-renewal ability in HSCs, which is caused by intrinsic harm to the HSCs^25^. The protection and improvement of HSCs’ self-renewal are at the core of the development of new anti-myelosuppressive drugs. To our knowledge, this is the first report on the effect of SIPS on hematopoiesis function, especially on HSCs/HPCs, and the underlying mechanisms. SIPS promoted the proliferation and differentiation of hematopoietic cells and improved the hemopoietic dysfunction induced by CTX in mice. SIPS is not a pure compound, which helps us explain the non-dose-dependent activities exhibited in these experiments. This is in fact a common feature of pharmaceutically active natural products^26^.

Hematopoiesis is actually a dynamically balanced process among the proliferation of HSCs, erythroid differentiation, and the formation of blood cells^27,28^. HSCs maintain the normal functioning of the entire hematopoietic system, which is regulated by a number of stimulatory hematopoietic regulatory factors^29^. CD235a is considered an erythroid-specific marker^30^, and CD41 is a well-known signature of megakaryocytes and platelets^31^. Using flow cytometry, we found that SIPS increased the fluorescence intensity of CD235a and CD41 in K562 cells, indicating its promotion of the differentiation of erythroid and megakaryocytic lineage cells, which was consistent with the benzidine staining results.

The synergistic effect of CD45 and B cell receptors helps activate B lymphocytes^32^. As a member of the Ig superfamily, CD19 is expressed in 98% of B220 + B lymphocytes and regulates the differentiation, maturation, and activation of B lymphocytes^33^. B lymphocytes, which are derived from HSCs and mature cells in the bone marrow, play an important role in maintaining the body’s normal humoral immunity. Once the immune system is out of balance, serious autoimmune diseases can be triggered^34^. In other words, abnormalities in the immune system can damage the hematopoietic system. In SIPS-treated mice with hematopoietic dysfunction, the percentage of CD19^+^CD45^+^ BMMNC cells was significantly increased. We also analyzed an additional two antigen markers associated with bone marrow hematopoiesis: sca-1 and c-kit. Sca-1 is a marker specific to mouse HSCs and is used to identify and isolate HSCs^35^. C-kit is expressed on the surfaces of both early and differentiated HSCs/HCPs and helps the proliferation and differentiation of cells^36^. Experiments have found that sca-1^+^ HSC/HPC transplantation can reconstruct the hematopoietic function of non-obese diabetic/severe combined immunodeficient mice^36^. Encouragingly, SIPS significantly enhanced the proportion of HSCs/HPCs in the BMMNCs of mice with hematopoietic dysfunction, further confirming its role in improving hematopoietic function.

The proliferation and differentiation of hematopoietic cells are regulated by soluble polypeptide-cytokines, including ILs, interferons, G-CSF, and erythropoietin^37–39^. IL-6 promotes the production of self-reactive antibodies by B lymphocytes, shows a clonogenic stimulating activity on HSCs and causes an increase in myelopoiesis^40^. Other ILs can synergistically promote the proliferation and differentiation of HSCs^41–43^. In particular, the activation of hematopoietic cells regulated by IL-3 and IL-5 is related to the activation of STAT3 and JAK2^44,45^. However, long-term chemotherapy/radiotherapy seriously damages the hematopoietic system and leads to the release of proinflammatory cytokines from host cells. The abnormal secretion of TNF-α and IFN-γ can directly inhibit the hematopoietic function of bone marrow by reducing the number of myeloid precursors and limiting the viability of HSCs^46^. In this sense, SIPS indeed regulated the levels of ILs, TNF-α, and IFN-γ, upregulated the contents of G-CSF and M-CSF, and decreased TNF-α and IFN-γ in the spleen and serum of mice with hematopoietic dysfunction. Key cytokines G-CSF, derived from osteoblasts, and M-CSF, derived from fibroblasts, can stimulate the proliferation and differentiation of bone marrow progenitor cells or neutrophilic precursor cells and enhance the myeloid commitment on the HSC level^47^. G-CSF is a pleiotropic cytokine that plays a major role as a regulator of hematopoiesis and an activator of innate immune responses, and it possesses protective effects against autoimmune neuroinflammatory diseases. G-CSF treatment in vivo can inhibit the secretion of TNF-α and IFN-γ by altering cytokine changes. However, the status of G-CSF in the immune system is regulated by the activity of immune cells, especially by increases in the number of circulating white blood cells. In addition, G-CSF can inhibit the apoptosis of neutrophils and increase the survival rate of neutrophils in infected tissues^48^. Mice that lack G-CSF or G-CSF receptor easily trigger chronic neutropenia, which is accompanied by a serious reduction in immature granulocytic precursors^49^. The combination of G-CSF with its cognate receptors was a susceptible trigger for the activation of the JAK/STAT pathway^21^. M-CSF plays crucial roles in the generation and characteristic differentiation of myeloid cells in bone marrow. M-CSF can increase the IL-4 level and reduce the TNF-α content in the experimental allergic encephalomyelitis model^50^. IFN-γ released from CD8 + cytotoxic T cells during a viral infection induced the secretion of IL-6 from BMMNCs^51^. Our results show a reduction of IFN-γ levels in serum and in the spleen and elevated IL-6 levels in mice with hematopoietic dysfunction after SIPS treatment for 28 days. IL-6-deficient mice have delayed hematopoietic recovery^52^, which indicates a role for IL-6 in the control of HSC proliferation and differentiation. Taken together with our data, the upregulated ILs and downregulated TNF-α and IFN-γ caused by SIPS treatment could be related to the higher levels of Treg cells present in the G-CSF mobilizing donor circulation. These results suggest that G-CSF could be an indispensable factor in SIPS’ protection against hematopoietic function.

G-CSF can regulate JAK2/STAT3 signaling, which helps to control hematopoietic cell proliferation and differentiation through a variety of growth factors^53,54^. STAT3 activation has been linked with both the differentiation and survival of myeloid cells^55^. Phosphor-JAK2 activates downstream signaling molecules, induces STAT3’s dimerization into nuclei, and then promotes the expression of related proliferating proteins to complete the proliferation and differentiation process^56^. Cytokines, including IL-2, IL-3, IL-4, and IL-6, can stimulate the tyrosine phosphorylation of JAK and activate STAT protein expression^57,58^. G-CSF can stimulate the JAK/STAT pathway in human choriocarcinoma cells, and a similar pattern of kinase phosphorylation was obtained in G-CSF-stimulated NFS-60 cells^59^. As reported, STAT3 seems to be involved in the IL-6–induced differentiation of monocytic cells in mice^60^. Our group previously confirmed that calf spleen extractive injection can improve CTX–induced hematopoietic injury by activating the G-CSF–mediated JAK/STAT pathway^61^. The results herein suggest that G-CSF plays a protective role in hematopoietic function. Similarly, we also found that SIPS stimulated the phosphorylation of the JAK2/STAT3 pathway and promoted the expression of functional transcription factors RSK1p90, c-Myc, and ETS transcription factor, which facilitate the self-renewal of HSCs.

In summary, we confirmed for the first time that SIPS protects against CTX–induced hematopoietic dysfunction via the G-CSF–mediated JAK2/STAT3 pathway. These findings support the theory that SIPS are an important source for screening of anti-myelosuppressive agents.

## Materials and methods

### Polysaccharides preparation

*S. imbricatus* fruiting bodies were dried and crushed, soaked in petroleum ether (1: 5, w/v) at 95 °C temperature for 2 h and then extracted with deuterium depleted (D.D.) water (1:15, w/v) for 3 h at 85 °C. This process was repeated three times. After concentration by a rotary evaporator under reduced pressure, the free proteins within the extracts were deproteinized using the Sevag method. The crude output of SIPS was precipitated with four times the volume of anhydrous ethanol at 4 °C for 24 h, collected using centrifugation (4000 rpm, 10 min, 4 °C) and lyophilized (Fig. s1). The polysaccharide extraction yield of *S. imbricatus* fruiting body is 16.4%, and the purity of SIPS is 86.8%.

### Cell culture

Human megakaryoblastic leukemia CHRF (CRL10107) and human myelogenous leukemia K562 (CCL-243™) cell lines were purchased from the American Type Culture Collection (ATCC; USA). The cells were cultured in a Roswell Park Memorial Institute (RPMI) 1640 medium with 15% fetal bovine serum (FBS, Gibco, Invitrogen, USA), 100 U/ml penicillin and 100 μg/ml streptomycin (Gibco, Invitrogen, USA) in a complete humidity incubator with 5%/95% CO_2_/air at 37 °C overnight. To ensure that the viability of the isolated cells was >95%, we used a Neubauer hemocytometer and the Trypan Blue dye exclusion method (Beyotime Biotech, Haimen, Jiangsu, China).

The primary bone marrow cells of male Balb/c mice (6–8 weeks old) were collected and flushed out from the femurs and tibias of each mouse using a 1 ml syringe with a 21-gauge needle containing Dulbecco’s Modified Eagle Medium (DMEM, Gibco, Invitrogen, USA). The red blood cell lysis buffer was used to removed non-nucleated cells from the collected bone marrow cells according to the product instruction manual. The resultant cells were washed and precipitated with phosphate-buffered saline (PBS). The cells were seeded at a density of 2 × 10^6^ cells/well into a 6-well plate in DMEM plus 10% FBS, 100 U/ml penicillin and 100 μg/ml streptomycin (Gibco, Invitrogen, USA). The cells were cultured in a complete humidity incubator with 5%/95% CO_2_/air at 37 °C.

### XTT assay

The XTT method was used to observe the effects of SIPS on the proliferation of CHRF and K562 cells according to previously reported methods^62^. In brief, 100 μl CHRF or K562 cells were seeded into 96-well plates at a concentration of 2 × 10^4^ cells/well and cultured in RPMI-1640 medium (contains 15% FBS). Subsequently, 100 μl of SIPS dissolved in non-FBS RPMI-1640 medium was added to each well and the final concentrations were kept at 0, 50, 100, and 200 μg/ml. After a 24-h incubation, 50 μl of a pre-warmed XTT-PMS application solution, which contained 50 μg of 2,3-bis-(2-methoxy-4-nitro-5-sulfophenyl)-2H-tetrazolium-5carboxanilide and 0.38 μg of phenazine methosulfate (Sigma-Aldrich), was added into each well. The cells were cultured for another 4 h. The absorbance was read at 450 nm on a microplate reader (Elx-800; Bio-Tek, USA).

### Erythroid differentiation of K562 cells

Benzidine staining was applied to analyze the erythroid differentiation and hemoglobin content of the K562 cells. 4 × 10^5^ of K562 cells were seeded into 6-well plates, and cultured with SIPS (dissolved in non-FBS RPMI-1640 medium) at concentrations of 0, 50, 100, and 200 μg/ml for another 24 h. The collected K562 cells were re-suspended with ice-cold PBS to 1 × 10^6^ cells/500 μl and mixed with 28 μl of benzidine solution and 2 μl of 30% hydrogen peroxide. After a 5 min reaction at room temperature, 2 μl of 5% solution of nitrosylated sodium cyanide was added. After another 15 min reaction, the cells were examined using a light-microscope digital camera (Nikon Instruments, Tokyo, Japan). The benzidine-positive cells were dark blue, while the benzidine-negative cells were light yellow. Groups of at least 200 cells were counted, and the proportion of positive cells in each group was calculated.

CD235a and CD41 was used to analyze the differentiation of the K562 to erythrocytic and megakaryocyte by flow cytometry, respectively. The K562 cells at a density of 4 × 10^5^ cells/ml were seeded into 6-well plates, and then co-cultured with SIPS (dissolved in non-FBS RPMI-1640 medium) at concentrations of 0, 50, 100, and 200 μg/ml for 48 h. The collected cell suspension (100 μl; containing 1 × 10^6^ cells) was incubated with PE-conjugated anti-human CD235a antibody (349106, Biolegend, USA) and the platelet membrane glycoprotein FITC-CD41 antibody (303704, Biolegend, USA) at room temperature in darkness for 15 min. The PE-conjugated anti-mouse IgG2a, κ (400213, Biolegend, USA) and the FITC-conjugated anti-mouse IgG1, κ (400107, Biolegend, USA) were set as the isotype controls. The expressions of CD235a and CD41 were analyzed on a Cytoflex flow cytometer (Beckman Coulter). Data were further analyzed using FlowJo software (Tree Star, Inc., OR, USA).

### Animal experiments and drug treatments protocol

Male BALB/c mice (4–6 weeks, 18–22 g, specific pathogen-free (SPF) grade; SCXK (JI)-2016-0003) were purchased from the lab animal center of Jilin University. The mice were housed in a controlled environment (temperature of 23 ± 1 °C and humidity of 50 ± 10%) with a 12/12 h light/dark cycle (lights on 8:00 a.m. to 8:00 p.m.). Food and tap water were given ad libitum. Our experimental protocol was approved by the Institution Animal Ethics Committee of Jilin University (No. 20160208).

After 1 week of acclimatization, 50 mice were intraperitoneally injected with CTX (100 mg/kg, Sigma-Aldrich, USA) dissolved in normal saline (NS) for 3 days to develop the hematopoietic dysfunction mouse model. Then the mice were divided randomly into five groups, which were treated orally with 10 ml/kg of double distilled (D.D.) water (model group; *n* = 10) once per day, SIPS (dissolved in D.D. water) at doses of 25 mg/kg (*n* = 10), 50 mg/kg (*n* = 10) and 100 mg/kg (*n* = 10) once per day, or subcutaneously injected with 22.5 μg/kg of rhG-CSF (Changchun Kinsey Pharmaceutical Co., Ltd.) twice a week for 4 weeks. To avoid the restoration of hematopoietic function in mice, CTX (80 mg/kg) was injected once a week. Another 20 mice were intraperitoneally injected with NS for 3 days and then orally treated with 10 ml/kg of D.D. water (control group; *n* = 10) and 50 mg/kg of SIPS (SIPS alone-treated group; *n* = 10) once per day for 4 weeks. All operations were conducted between 9:00 and 11:00 a.m. Mice were weighed on day 1, 4, 11, 18, and 28. Two hours after the last agent was administered, blood was sampled from the caudal veins, and the changes of peripheral blood were analyzed immediately with a fully automatic blood analyzer (Drew Scientific Group, Dallas, TX). All mice were killed by injecting 200 mg/kg of pentobarbital. The spleen, thymus, liver, and kidney tissues were immediately collected and weighed. The organ indexes (%) were calculated as follows: organ index (%) = organ weight (mg)/bodyweight (g).

### Isolation of BMMNCs and surface antigen analysis

The experimental mice were euthanized, and then the femoral and tibial parts were immediately removed under sterile conditions. DMEM-low glucose (Gibco, Invitrogen, USA) containing 1% (vol/vol) penicillin/streptomycin (Gibco, Invitrogen, USA) was used to wash out the bone marrow components using a syringe with a 21-gauge needle. After removing the small pieces of bone and debris, the BMMNCs were isolated with an ACK lysis buffer and adjusted to 1 × 10^6^ cells/100 μl. The activity of the cells was detected by Trypan Blue staining ( > 95%).

The antibodies of surface markers including PerCP-conjugated anti-mouse CD45 (103129), APC-conjugated anti-mouse CD19 (152410), FITC-conjugated anti-mouse Lineage Cocktail (133302), PE-conjugated anti-mouse Ly-6A/E (sca-1; 108108), and APC-conjugated anti-mouse c-kit (135108) were used to exclude HSCs and leukocytes, which were stained with 100 μl cells for 15 min at room temperature in the darkness. PerCP-conjugated anti-rat IgG2b (400629), APC-conjugated anti-rat IgG2b (400611), FITC-conjugated anti-rat IgG2b (400605), PE-conjugated anti-mouse IgG2a (400213) and FITC-conjugated anti-rat IgG2a (400505) were set as isotype controls. The cells were finally analyzed using a Cytoflex flow cytometer (Beckman Coulter). All antibodies were obtained from Biolegend (San Diego, CA).

### Histopathological analysis

The collected femoral condyle, spleen, liver, and kidney were fixed in a neutral buffered 10% formalin solution for 48 h to maintain the original morphological structure of cells. Subsequently, the tissues were embedded in paraffin and sliced to a thickness of 5 μm according to previous methods^63^. In particular, the bones were decalcified for 7 days. All slides were analyzed using H&E staining and detected under the inverted microscope CKX41 (Olympus, Japan).

### High-throughput splenic antibody chip analysis

Cytokines and chemokines are extracellular signaling molecules that mediate cell–cell communication. The Mouse Cytokine Array Panel A Kit (R&D Systems, Minneapolis, MN) was used to analyze the 40 different cytokines/chemokines of mouse spleen. According to the manufacturer’s instructions, the spleen tissues were homogenized in PBS with protease inhibitors (10 μg/ml Aprotinin, 10 μg/ml Leupeptin, and 10 μg/ml Pepstatin A; Sigma-Aldrich, USA). After adding 1% triton X-100 (Sigma, Catalog #T9284), the samples were centrifuged at 10,000 × *g* for 5 min to remove cellular debris. The protein concentrations were analyzed using a bicinchoninic acid (BCA) protein assay kit (Merck Millipore, Billerica, MA). The reconstituted Mouse Cytokine Array Panel A Detection Antibody Cocktail (15 μl) was added to prepared protein samples and incubated at room temperature. The membranes contained 40 different cytokine antibodies were pre-blocked with bovine serum albumin (BSA) for 1 h. The antibodies bound to the membrane antibodies were detected using the diluted Streptavidin-HRP and Chemi Reagent Mix. The membranes were then exposed using a LI-COR Odyssey Scanner (LI-COR Corporate, USA). The ProtoArray® Prospector software (Life Technologies, Carlsbad, CA, USA) was used to analyze the data.

### Detection of cytokines in serum and spleen

Cytokines/chemokines including IL-1Ra, IL-2, IL-3, IL-4, IL-5, IL-6, G-CSF, M-CSF, TNF-α, IFN-γ, CCL1, and MCP-1 in serum and spleen collected from the experimental mice were measured using ELISA kits (Shanghai Yuanye Bio-Technology Co., Ltd. Shanghai, China).

### Western blotting

The CHRF and K562 cells were seeded into 6-well plates at a density of 5 × 10^5^ cells/well, and the BMMNCs were seeded into 6-well plates at a concentration of 2 × 10^6^ cells/well. The cells were incubated with SIPS for 24 h. The proteins from the treated cells and spleen of the experimental mice were collected using a radio-immunoprecipitation assay lysis buffer containing 1% protease inhibitor cocktail (Sigma-Aldrich, USA) and 2% phenylmethanesulfonyl fluoride (Sigma-Aldrich, USA) and the concentration was analyzed using a BCA protein assay kit. The protein (40 μg) was separated by 12% sodium dodecyl sulfate polyacrylamide gel electrophoresis (SDS-PAGE) and transferred onto a polyvinylidene difluoride membranes (0.45 μm, Merck Millipore, Billerica, MA). The membranes were blocked at room temperature with 5% BSA for 2 h and then incubated with primary antibodies (diluted to 1:2000). The antibodies included RSK1p90 (ab32526), phosphor(P)-RSK1p90 (ab32413), c-Myc (ab32072), ELK1 (ab188316), G-CSF (ab181053), G-CSFR (ab19479), JAK2 (ab108596), phosphor (P)-JAK2 (ab32101), STAT3 (ab119352), phosphor (P)-STAT3 (ab76315; Abcam, Cambridge, MA), M-CSF (sc-365779; Santa Cruz Biotechnology, Santa Cruz, CA), and GAPDH (ABS16; Millipore, Merck Millipore, Billerica, MA) and were incubated overnight at 4 °C. After washes with a tris-buffered saline buffer containing 0.1% Tween-20, the membranes were exposed to horseradish peroxidase (HRP)-conjugated secondary antibodies (diluted to 1:4000) for 2 h at room temperature. The corresponding protein bands were developed using an enhanced chemilumenescent detection kit (Merck Millipore, Billerica, MA) and visualized with an imaging system (BioSpectrum600). The pixel density was quantified using the ImageJ software (National Institutes of Health, Bethesda, MD).

### Statistical analysis

Statistical significance was determined using a one-way ANOVA followed by a Dunnett’s post hoc comparison using SPSS 16.0 software (IBM Corporation, Armonk, NY). All values are expressed as mean ± S.D. Results were considered significant at *P* < 0.05.

## Electronic supplementary material

Figure S1

Figure S2

Figure S3

Supplementary figure legends
