# Supplementary figures and images for: Sarcodon imbricatus polysaccharides improve mouse hematopoietic function after cyclophosphamide-induced damage via G-CSF mediated JAK2/STAT3 pathway
Source: Cell Death Dis. 2018 May 21;9(6):578. doi: 10.1038/s41419-018-0634-6 (PMC5962553; doi:10.1038/s41419-018-0634-6)

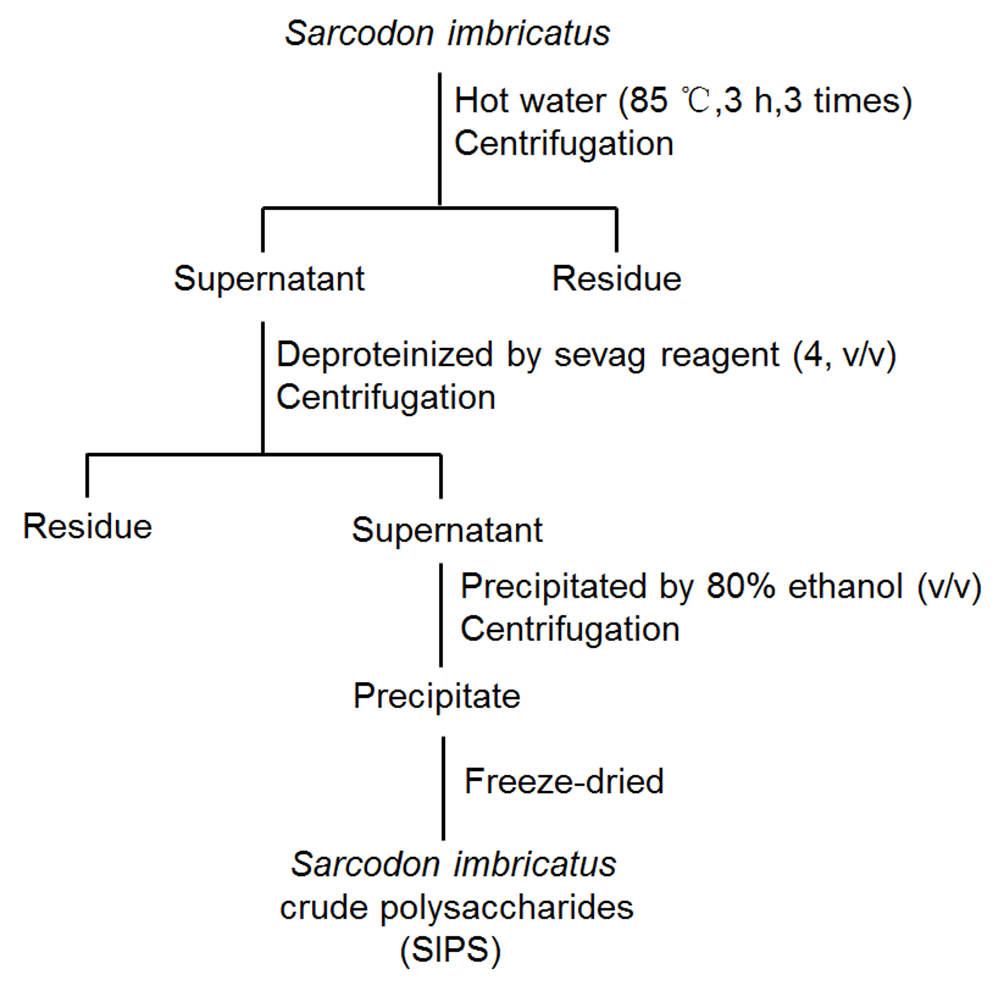

Supplement: Supplementary file 1 — Figure S1 [file 41419_2018_634_MOESM1_ESM.tif]

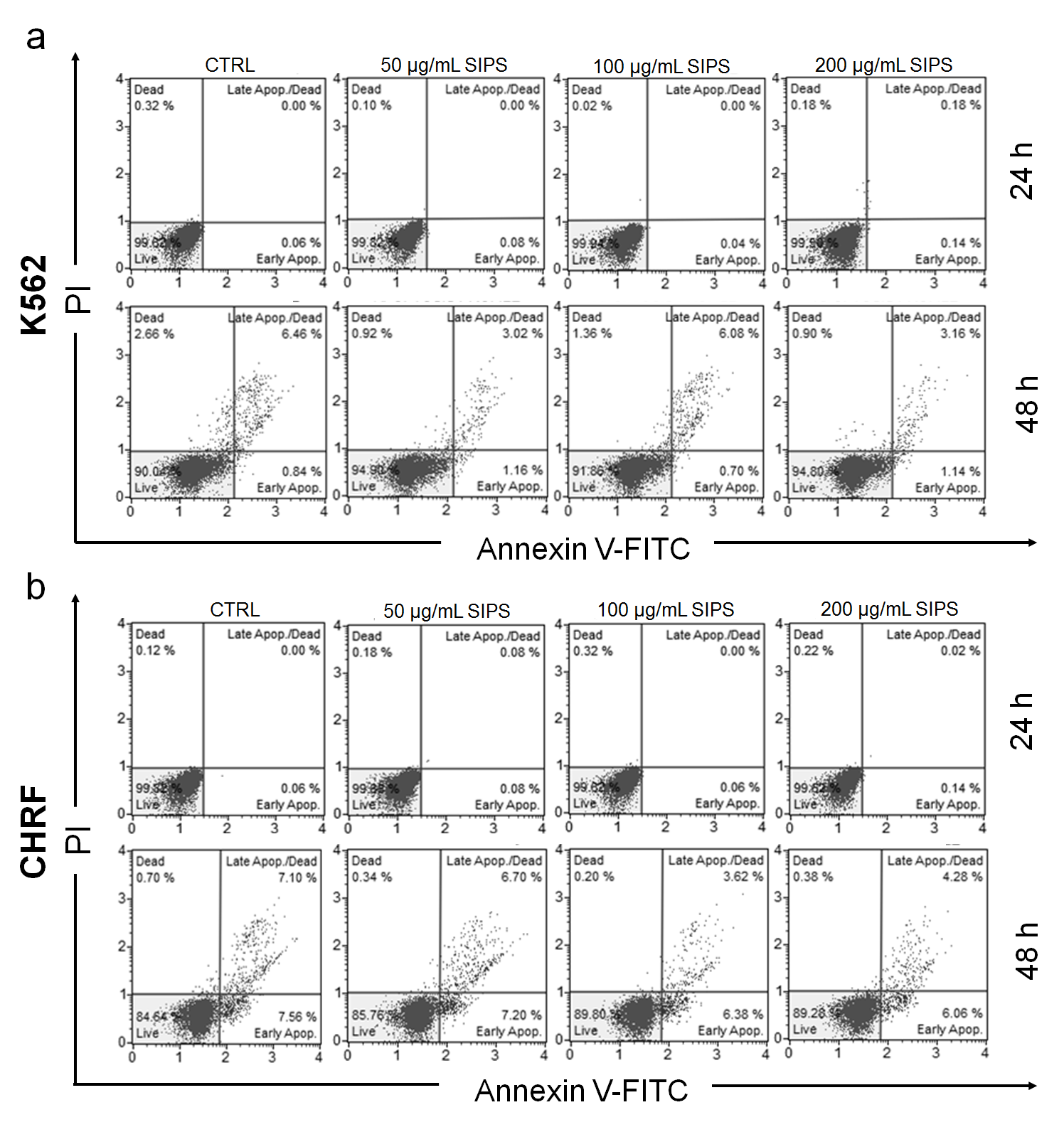

Supplement: Supplementary file 2 — Figure S2 [file 41419_2018_634_MOESM2_ESM.tif]

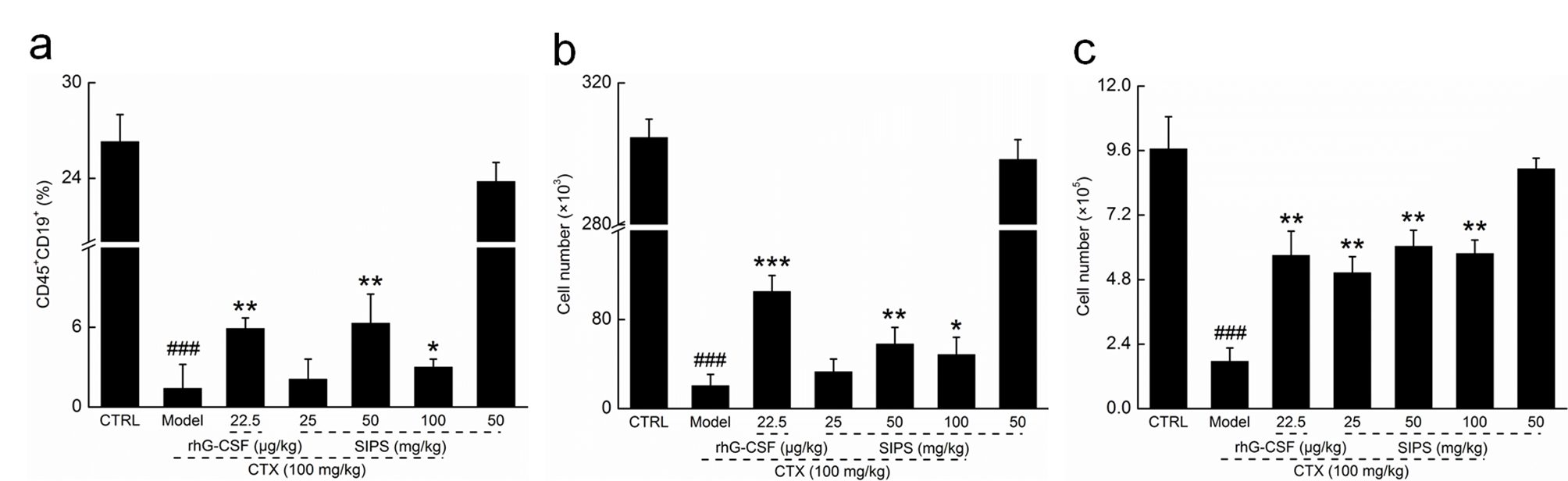

Supplement: Supplementary file 3 — Figure S3 [file 41419_2018_634_MOESM3_ESM.tif]
