# Supplementary material for: Sarcodon imbricatus polysaccharides improve mouse hematopoietic function after cyclophosphamide-induced damage via G-CSF mediated JAK2/STAT3 pathway
Source: Cell Death Dis. 2018 May 21;9(6):578. doi: 10.1038/s41419-018-0634-6 (PMC5962553; doi:10.1038/s41419-018-0634-6)
Supplement: Supplementary file 4 — Supplementary figure legends [file 41419_2018_634_MOESM4_ESM.docx]

**Figure list**

**Fig.S1.** Schematic illustration of *Sarcodon imbricatus* crude polysaccharides extraction and separation

**Fig.S2.** Effect of SIPS on cell apoptosis of K562 and CHRF. The K562 and CHRF cells were co-cultured with SIPS (0, 50, 100, 200 µg/ml) for 24 h and 48 h, and cell apoptosis of K562 and CHRF was detected by Annexin V/PI staining via flow cytometry (n=6).

**Fig.S3.** The B lymphocytes (**a**) expression in mice bone marrow cells. And the absolute number of HSCs (**b**) and HPCs (**c**) of per mouse (from 2 tibiae and 2 femora) after 28-day administration of SIPS or rhG-CSF. Data are expressed as the means ± S.D. (n=10). ^###^ *P* < 0.001 vs. the control group, * *P* < 0.05, ** *P* < 0.01 and *** *P* < 0.001 vs. the model group.
